# Linalool May Exert Neuroprotective Effects Against Cadmium-Induced Hippocampal Neurodegeneration by Regulating the 4-HNE/NF-κB Signaling Pathway

**DOI:** 10.1007/s12011-025-04734-7

**Published:** 2025-07-05

**Authors:** Sercan Kaya, Tuba Yalçın, Devran Ayyıldız, Buşra Akyol

**Affiliations:** Vocational Higher School of Healthcare Studies, Health Services Vocational School, Batman University, Room 212, Kültür Neighborhood, Batman, Turkey

**Keywords:** Cadmium, Linalool, Hippocampus, 4-hydroxynonenal, Nuclear factor kappa B

## Abstract

Cadmium (Cd) is a prevalent industrial and environmental contaminant that accumulates in the tissues of animals and humans. In particular, one of the regions most affected by Cd-induced neurodegeneration is the hippocampus. Linalool (Lin) is a monoterpene capable of traversing the blood–brain barrier owing to its low molecular weight and high lipophilicity. This study sought to establish the possible neuroprotective effects of Lin against Cd-induced hippocampal neurodegeneration by focusing on the 4-hydroxynonenal (4-HNE)/Nuclear factor kappa B (NF-κB) signaling pathway. The 28 male rats used in the experiment were randomly (*n* = 7) distributed into four groups. The control group was not administered. The Cd group received CdCl_2_ at a dose of 3 mg/kg (cumulative 21 mg/kg) for the first 7 days of the experiment. The Cd + Lin group was given 3 mg/kg CdCl_2_ for the first 7 days of the experiment and 100 mg/kg Lin for 14 days throughout the experiment. The Lin group was administered Lin at a dosage of 100 mg/kg/day. After all treatments were finished, the rats’ brain tissues were taken out on the 15th day of the experiment. In this study, Cd increased oxidative stress parameters and decreased antioxidant enzyme levels in hippocampus tissue. In addition, Cd caused an increase in NF-κB and pro-apoptotic Caspas3 levels, which play an important role in inflammation in the hippocampus. Lin treatment against Cd exposure showed a therapeutic effect by suppressing oxidative stress, inflammation, and apoptosis. Lin may exert a neuroprotective effect by regulating the 4-HNE/NF-κB signaling pathway in Cd exposure-induced hippocampal neurodegeneration.

**Graphical Abstract:**

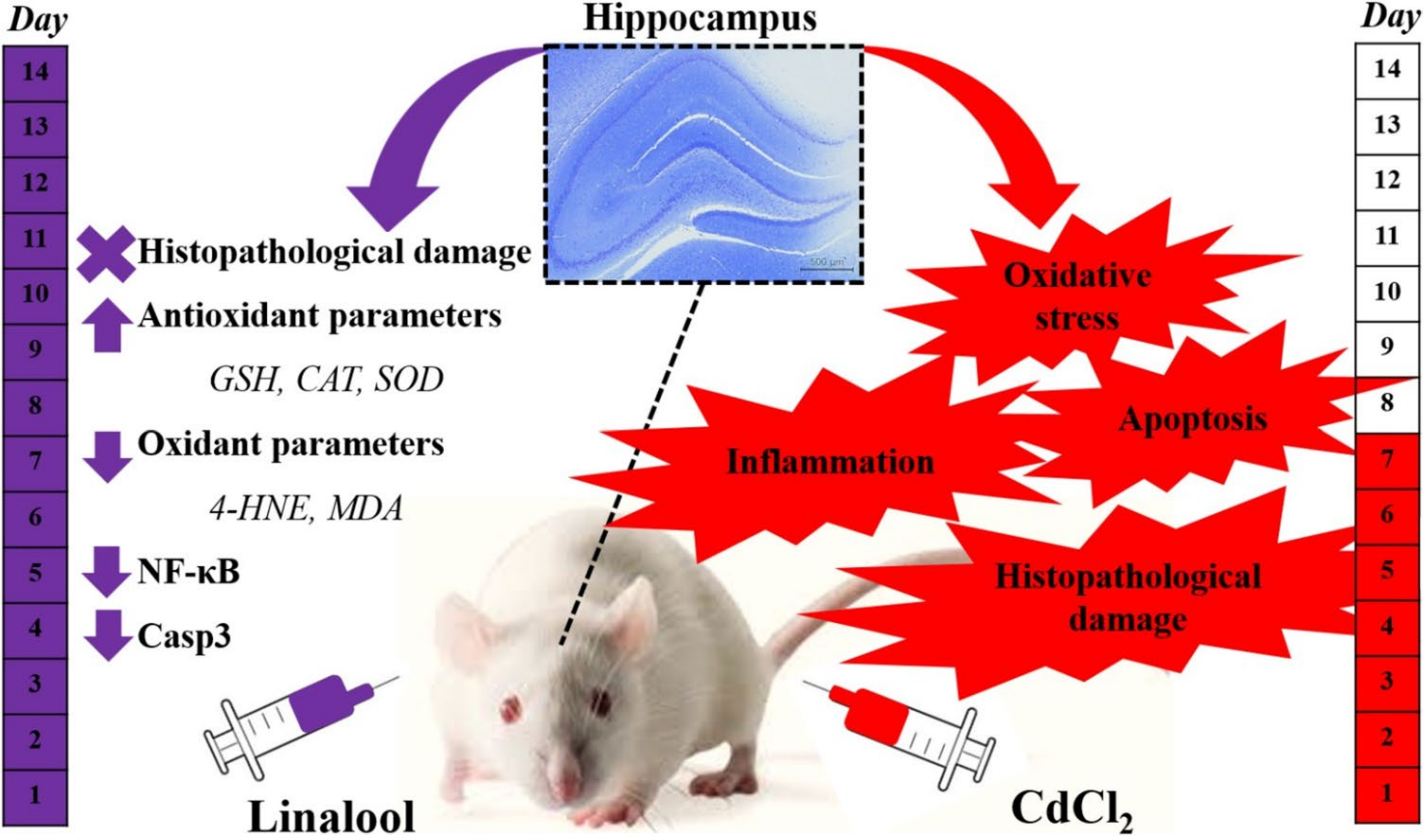

## Introduction

Cadmium (Cd) is a prevalent industrial and environmental contaminant that accumulates in the tissues of animals and humans [1]. Metal industries, the manufacture of some batteries, consuming tainted food or water, smoking tobacco, or breathing in contaminated air are the main ways that people might be exposed to Cd [2]. Numerous tissues have been shown to sustain structural and functional damage as a result of Cd exposure [3]. The kidney, lung, gastrointestinal tract, bone, and central nervous system are among the organs that can be harmed by Cd toxicity [2]. Symptoms of Cd accumulation in the brain include headache, olfactory impairment, peripheral neuropathy, decreased vasomotor function, decreased ability to concentrate, loss of balance, and learning difficulties [4]. Cd concentration has been shown to be associated with reduced motor skills and mental retardation in humans [5]. It has been documented that exposure to Cd in the brain results in toxicity and cell death in cortical neurones. Cd increases the production of reactive oxygen species (ROS), which worsen neuronal inflammation and apoptosis, while suppressing the antioxidant defence system and causing mitochondrial malfunction [6]. Thus, oxidative stress brought on by Cd damages neurones in several brain regions both structurally and functionally. According to studies, Cd alters the structure and function of many brain areas, resulting in neurodegeneration [7, 8]. In particular, the hippocampus suffers from marked Cd-induced neurodegeneration [9]. Since it triggers an inflammatory response that is verified by neuronal death with reactive astrocytosis and apoptosis seen exclusively in hippocampus neurones, Cd has been implicated as a factor that induces neurodegeneration [10]. One study showed that Cd exposure caused a decrease in dendritic branching and density in the hippocampal regions of Dentate gyrus (DG), Cornu ammonis (CA)1 and CA3 [8]. In a similar vein, a different study has demonstrated that Cd exposure induces a severe oxidative response that significantly contributes to hippocampal [9].

ROS such as O^−2^ and H_2_O_2_ cause peroxidation reactions in unsaturated fatty acids in cell membranes, affecting their structural integrity and resulting in cellular dysfunction. Lipid peroxidation is a significant aspect of Cd-induced oxidative stress [11]. Malondialdehyde (MDA) and 4-hydroxynonenal (4-HNE), two extremely toxic and reactive byproducts of this process, can react with proteins, lipids, and nucleic acids to generate persistent adducts that further impair cellular function [12]. Abnormal accumulation of toxic end-products leads to neurodegeneration through the activation of biochemical and metabolic pathways that disrupt the structure and function of the brain [13]. For instance, one study found that brain tissue MDA levels increased after Cd exposure [14]. 4-HNE, another lipid peroxidation product, has been reported to form adducts with proteins, affect their biological functions, and disrupt intracellular homeostasis [15]. Therefore, 4-HNE and MDA can be used as indicators of the severity of lipid damage produced by free radicals.

Antioxidant-effective molecules can prevent oxidation-induced damage in the cellular base by preventing the formation of reactive oxygen or scavenging the active oxygen [16]. Suppression of oxidative stress in living metabolism by antioxidants helps to protect lipids, proteins, and cell integrity by preventing lipid peroxidation [17, 18]. Because of their diverse bioactivities, natural products like essential oils and their constituents are frequently employed [19]. A monoterpene called linalool (Lin) is a constituent of several essential oils that are derived from plants including lavender and cardamom [20]. These substances’ high lipophilicity and low molecular weight allow them to pass through the blood–brain barrier. As a result, they can be utilized in the pharmacological management of cognitive and behavioral abnormalities [21]. ROS stimulates a transcription factor called nuclear factor kappa B (NF-κB), which is present in cells and is crucial for the transcription of genes related to inflammation [22]. It has been reported that Lin shows an anti-inflammatory effect by inhibiting inflammatory signals produced by NF-κB. It has also been reported that Lin may suppress oxidative stress by reducing ROS and cytokine production [23, 24]. According to recent research, Lin has anti-inflammatory and antioxidant properties that protect the kidney [1], liver [19], and testicular [20] tissues against Cd-induced damage. In addition, studies have shown that Lin has anti-inflammatory and antioxidant effects as well as neuroprotective effects [23]. One study, for instance, determined that Lin decreased neuronal cell death by increasing mitochondrial respiration in a neuronal cell line (HT-22) and rodent hippocampal tissue as a defense mechanism against glutamate hyperstimulation [25]. Different in vitro studies have shown that Lin reduced TNF-induced inflammation in brain endothelial cells [26] and oxidative damage due to hydrogen peroxide exposure in neuronal (SH-SY5Y) cells [27]. A study on the Alzheimer’s (3xTg-AD) triple transgenic mouse model revealed that Lin treatment reduced amyloid burden, neuroinflammation, and tauopathy in the amygdala and hippocampus [28]. Another study reported that Lin prevented oxidative stress in brain tissue caused by chronic D-galactose and aluminum trichloride administration [29]. In addition, Lin administration was shown to reduce infarct volume and suppress inflammatory markers in the cortex and hippocampus in the ischemia rat model (MCAOR) [30]. The restoration of lipid homeostasis in the hippocampus was one of the methods by which Lin therapy was demonstrated to enhance neurological scores in the MCAOR rats ischemia model in another investigation [31].

Cd exposure-induced oxidative stress, especially in hippocampal neurons, and inflammatory response resulting in neuronal apoptosis cause neurodegeneration. By concentrating on the 4-HNE/NF-κB signalling pathway, this study sought to ascertain the possible neuroprotective effects of Lin, which has demonstrated anti-oxidant and anti-inflammatory actions against Cd-induced hippocampus neurodegeneration.

## Materials and Methods

### Experimental Design

The study was initiated with the approval of Fırat University Animal Experiments Ethics Committee dated 06.05.2024 and numbered 23996 (annex: 20.03.2025–33170). All experimental procedures were performed in accordance with ARRIVE guidelines. Cd (CdCl_2_, 99.99%, 20290810108–64-2, Sigma-Aldrich) and Lin (97%, L2602-102524372, Sigma-Aldrich) used in the experiment were purchased from commercial companies.

The 28 male Sprague–Dawley rats, weighing 220 ± 20 g and aged 8–10 weeks, were preserved in ideal conditions, which included perpetual water and food, a 12-h day/night light cycle, and 23 ± 2 °C. Rats were randomly distributed to the study groups in equal numbers.

***Control (n = 7),*** rats in this group were given only standard rat chow and water.

***Cd (n = 7),*** rats in this group were given CdCl2 (cumulative 21 mg/kg) at a dose of 3 mg/kg for the first 7 days of the experiment (week 1) and not administered for the last 7 days of the experiment.

***Cd + Lin (n = 7),*** rats in this group received CdCl_2_ at a dose of 3 mg/kg for the first 7 days (week 1) and Lin at a dose of 100 mg/kg throughout the experiment (14 days).

***Lin (n = 7),*** rats in this group received Lin at a dose of 100 mg/kg every day during the 14-day experiment.

Lin and Cd (Lin was dissolved in saline at 100 mg/1 ml and CdCl2 at 1 mg/1 ml) were administered intraperitoneally. The experimental design’s Cd and Lin dosages and uses were based on earlier research [32, 33]. Following the completion of all administrations, the rats were decapitated under ketamine/xylazine anesthesia on the 15th day of the trial, and the brain tissues were extracted.

### Histopathologic Evaluation

After completion of the experiment, the brain tissues obtained were subjected to cold chain. The right hemisphere of the brain was dissected and placed in 10% neutral formalin fixative for histopathological and immunohistochemical evaluations. After fixation, brain tissues were subjected to histologic follow-up series and paraffin blocks. Cresyl violet staining procedure was applied to the Sects. (5 µm thickness) taken from the blocks [34]. The slides were examined under an optical microscope and photomicrographs were taken (Leica, DM2500/MC170 HD, Germany). Ten randomly selected non-overlapping areas at × 40 magnification were evaluated to score histopathologic changes. Hippocampus Damage Score (HDS, % shrunken cells) pyramidal neurons were scored as 0: < 5%, 1: < 25%, 2: < 50%, 3: < 75%, and HDS table was created [35].

### Immunohistochemical Evaluation

4-HNE (ab46545, Abcam, UK), NF-κB (AF5006, Affinity, USA) and Casp3 (AF6311, Affinity, USA) immunoreactivities in brain (right) tissues were determined by avidin–biotin-peroxidase complex method using the procedure described previously [36]. All sections were counterstained with Mayer hematoxylin. Sections were examined under an optical microscope and photomicrographs were taken (Leica, DM2500/MC170 HD, Germany). 4-HNE, NF-κB and Casp3 immunoreactivities in the hippocampal area were determined by evaluating 10 different non-overlapping areas at × 20 magnification from each section. Immunoreactivities were computed using the immunostaining prevalence X immunostaining severity formula [37].

### Biochemical Analysis

After the completion of the experiment, the brain tissues obtained were subjected to a cold chain. The hippocampal tissues that were dissected from the brain’s left hemisphere were kept at − 80 °C. Hippocampus tissues were thawed once under appropriate conditions, and homogenates were obtained. Briefly, hippocampus tissues were homogenized using a Bullet Blender homogenizer device, to which cold phosphate buffered saline (pH:7.4) was added twice its weight. After centrifugation (+ 4 °C, 20 min, 3000 rpm), supernatants were obtained from the hippocampus tissue samples. NF-κB (BT Lab., China), Glutathione (GSH) (BT Lab., China), Catalase (CAT) (FineTest, China), Superoxide Dismutase (SOD) (FineTest, China), and MDA (FineTest, China) levels were determined using rat-specific ELISA kits according to the manufacturer's procedures.

### Statistical Analysis

Statistical analysis of the data obtained in the study was performed in SPSS 21.0 package program. The normal distribution of the study data was checked by Shapiro–Wilk test. Normally distributed parameters were analyzed using one-way ANOVA post hoc Tukey tests, and the results were presented as mean ± standard deviation. For the statistical analysis of non-normally distributed parameters, Kruskal–Wallis followed by Mann–Whitney *U* tests were used, and the results were presented as median (minimum–maximum). As a result of statistical analysis, *p* < 0.05 was defined as a significant difference.

## Results

### Effect of Lin Treatment on Cd-Induced Hippocampal Damage

**Fig. 1:**
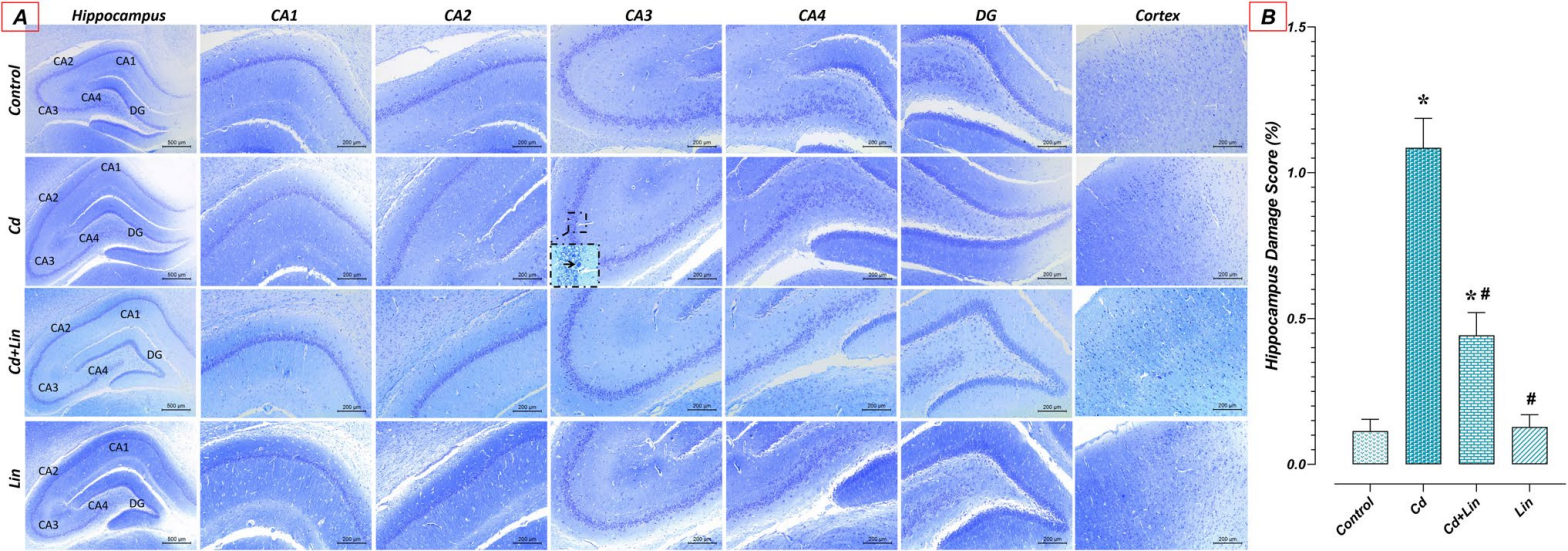
Effect of Cd exposure and Lin treatment on hippocampus and cerebral cortex. Hippocampus and cortex were normal histologically in the control and Lin groups. Cellular structures showing neodegeneration were observed in the Cd group compared to the control group. In the Cd+Lin group, compared to the Cd group, the cellular structures showing neodegeneration were significantly reduced. Arrow: pyramidal shrunken cells. A:Cresyl violet staining; scale bar 500 μm (hippocampus), 100 μm (CA 1-4, DG, Cortex) B: Hippocampus damage score graph. CA1-2-3-4; Cornu Ammonis 1-2-3-4, DG; Dentate gyrus, Cd; Cadmium, Lin; Linalol

The hippocampus (Cornu ammonis (CA) 1–4, Denta giyrus) and cerebral cortex structures were similarly normal histologically in the control (*n* = 7) and Lin (*n* = 7) groups. Shrunken cells showing dystrophic histopathologic changes such as abnormal Nissl granule distribution, shrunken hyperchromatic, and irregular chromatolysis were raised in the Cd group (*n* = 7) compared to the control group. However, Cd-induced histopathologic changes in the hippocampus were significantly reduced in the Cd + Lin group (*n* = 7) compared to the Cd group (Fig. 1).

Similarly, histopathologic examination revealed that HDS was similar between the control (*n* = 7) and Lin (*n* = 7) groups (*p* > 0.05). Cd (*n* = 7) exposure significantly increased HDS compared to the control group (*p* < 0.05). However, HDS decreased significantly in the Cd + Lin group (*n* = 7) compared to the Cd group (*p* < 0.05) (Fig. 1).

### Effect of Lin and/or Cd Applications on Antioxidant Parameters in Hippocampus

**Fig. 2:**
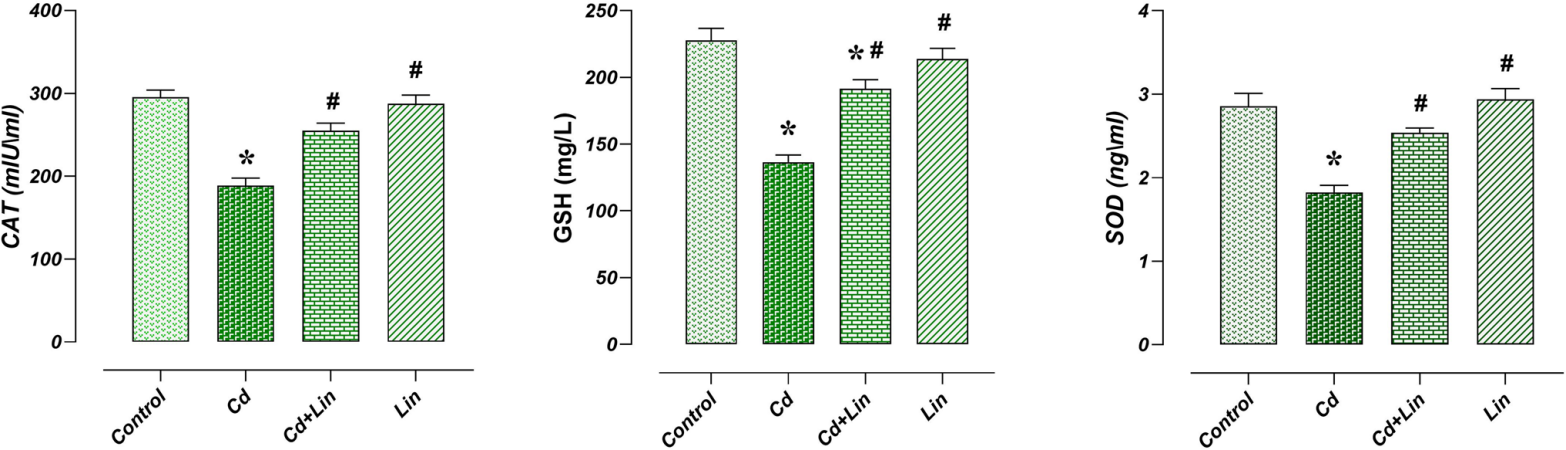
Effect of Lin and/or Cd applications on antioxidant parameters in hippocampus. CAT, SOD and GSH levels were similar in the control and Lin groups. When the Cd group was compared with the control group, CAT, SOD and GSH levels decreased. In Cd+Lin group, CAT, SOD and GSH levels were increased compared to Cd group. *; compared to control group (p < 0.05), #; compared to Cd group (p < 0.05). A; CAT levels, B; SOD levels, C; GSH levels, CAT; Catalase, SOD; Superoxide dismutase, GSH; Glutathione, Cd; Cadmium, Lin; Linalol

CAT, SOD, and GSH levels, which are important antioxidants in hippocampus tissue, were found to be similar in control (*n* = 7) and Lin (*n* = 7) groups. The Cd group (*n* = 7) had lower levels of CAT, SOD, and GSH than the control group (*p* < 0.05). However, compared to the Cd group, the Cd + Lin group (*n* = 7) had significantly higher hippocampus CAT, SOD, and GSH levels (*p* < 0.05) (Fig. 2).

### Effect of Lin and/or Cd Treatments on 4-HNE and MDA Levels in the Hippocampus

**Fig. 3:**
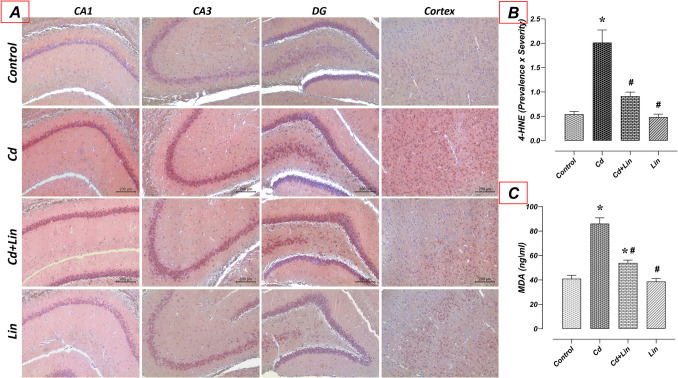
Effect of Lin and/or Cd treatments on 4-HNE and MDA levels in the hippocampus. Hippocampal 4-HNE and MDA levels were similar in the control and Lin groups. 4-HNE and MDA levels were increased in the Cd group compared to the control group. In the Cd+Lin group, 4-HNE and MDA levels were decreased compared to the Cd group. A: 4-HNE immunohistochemical staining microphotographs (scale bar; 200μm), B: 4-HNE immunoreactivity graph, C: MDA hippocampal ELISA levels. MDA; malondialdehyde, 4-HNE; 4-hydroxynonenal, CA1-2-3; Cornu Ammonis 1-2-3, DG; Dentate gyrus, Cd; Cadmium, Lin; Linalol

4-HNE and MDA levels in hippocampus tissue were similar in control (*n* = 7) and Lin (*n* = 7) groups. Compared to the control group, 4-HNE immunoreactivity and hippocampal MDA levels raised in the Cd group (*n* = 7) (*p* < 0.05). However, 4-HNE immunoreactivity and hippocampal MDA levels reduced in the Cd + Lin group (*n* = 7) compared to the Cd group (*p* < 0.05) (Fig. 3).

### Effect of Lin and/or Cd Treatments on NF-κB Levels in the Hippocampus

**Fig. 4:**
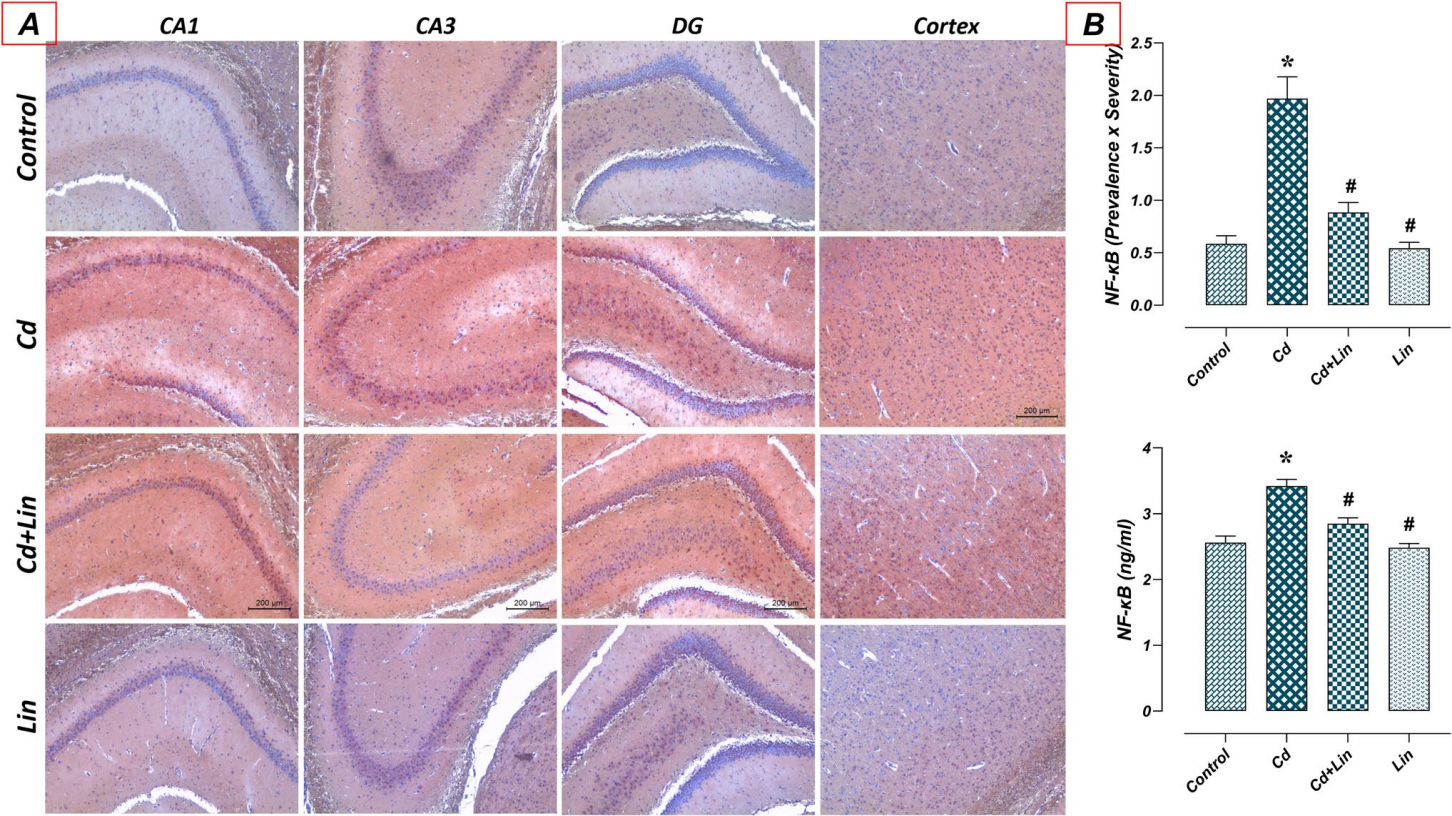
Effect of Lin and/or Cd treatments on NF-κB levels in the hippocampus. Hippocampal NF-κB levels were similar in the control and Lin groups. In the Cd group, NF-κB levels increased compared to the control group. In the Cd+Lin group, there was a decrease in NF-κB levels compared to the Cd group. A: NF-κB immunohistochemical staining microphotographs (scale bar; 200μm), B: NF-κB immunoreactivity graph, C: NF-κB hippocampal ELISA levels. NF-κB: Nuclear factor kapa B, CA1-2-3; Cornu Ammonis 1-2-3, DG; Dentate gyrus, Cd; Cadmium, Lin; LinaloolEffect of Lin and/or Cd treatments on NF-κB levels in the hippocampus. Hippocampal NF-κB levels were similar in the control and Lin groups. In the Cd group, NF-κB levels increased compared to the control group. In the Cd+Lin group, there was a decrease in NF-κB levels compared to the Cd group. A: NF-κB immunohistochemical staining microphotographs (scale bar; 200μm), B: NF-κB immunoreactivity graph, C: NF-κB hippocampal ELISA levels. NF-κB: Nuclear factor kapa B, CA1-2-3; Cornu Ammonis 1-2-3, DG; Dentate gyrus, Cd; Cadmium, Lin; Linalool

In the study, NF-κB immunoreactivity and ELISA levels in the hippocampus tissue of control (*n* = 7) and Lin (*n* = 7) groups were similar. Hippocampal NF-κB levels raised in the Cd group (*n* = 7) compared to the control group (*p* < 0.05). In the Cd + Lin group (*n* = 7), hippocampal NF-κB levels were reduced compared to the Cd group (*p* < 0.05) (Fig. 4).

### Effect of Lin and/or Cd Applications on Casp3 Immunoreactivity in the Hippocampus

**Fig. 5:**
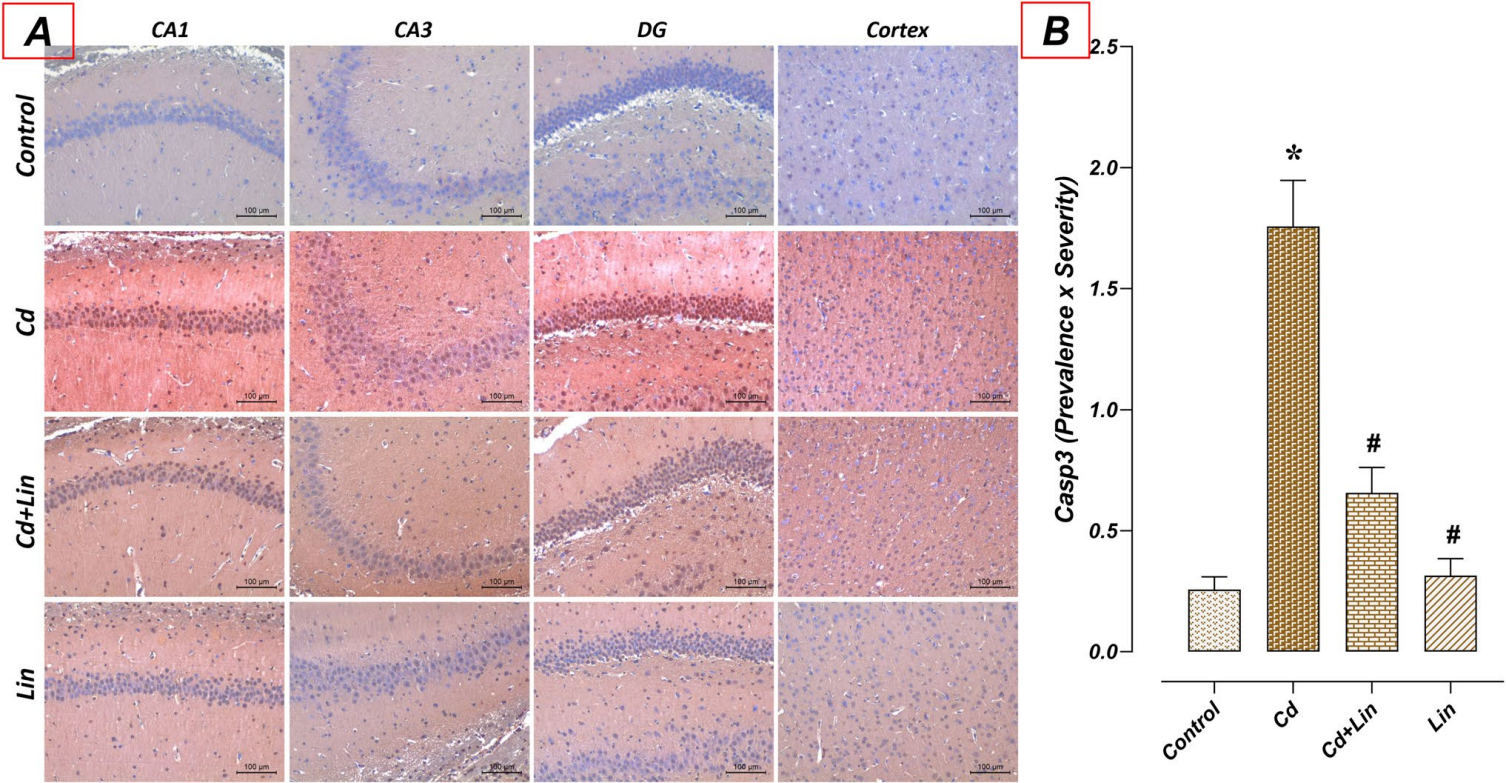
Effect of Lin and/or Cd applications on Casp3 immunoreactivity in the hippocampus. Casp3 immunoreactivities in the control and Lin groups were similar. Casp3 immunoreactivity was increased in the Cd group compared to the control group. However, Casp3 immunoreactivity was significantly decreased in the Cd+Lin group compared to the Cd group. A: Casp3 immunohistochemical staining microphotographs (scale bar; 100μm), B: Casp3 immunoreactivity graph. Casp3: Caspase 3, CA1-2-3; Cornu Ammonis 1-2-3, DG; Dentate gyrus, Cd; Cadmium, Lin; Linalol

In the study, Casp3 immunoreactivity in the hippocampus tissue of the control (*n* = 7) and Lin (*n* = 7) groups was at similar levels. When compared to the control group, Casp3 immunoreactivity was found to be significantly increased in the Cd group (*n* = 7) (*p* < 0.05). However, compared to the Cd group, hippocampus Casp3 immunoreactivity was significantly decreased in the Cd + Lin group (*n* = 7) (*p* < 0.05) (Fig. 5).

## Discussion

Humans and other living things are exposed to Cd, a heavy metal that has hazardous effects. Cd’s capacity to penetrate the blood–brain barrier, induce oxidative stress, and trigger the release of pro-inflammatory cytokines are among its primary harmful effects [38]. Although the most effective way to recover from Cd exposure is to minimize exposure, this is not always possible. Therefore, this study focused on the possible therapeutic effects of Lin against Cd-induced neurodegeneration. Lin is a low molecular weight, extremely lipophilic substance that has lately demonstrated anti-inflammatory and antioxidant properties. Lin has been shown to enter the bloodstream and/or brain and change neurochemical and neurotrophic signaling in many brain regions, while its exact methods of action are still unclear. Lin influences the brain’s oxidative stress, neurotrophic, and inflammatory pathways in addition to dopamine, glutamate, serotonin, and acetylcholine [23]. In this study, we report that Lin may exert a neuroprotective effect against Cd-induced neurodegeneration by suppressing oxidative stress, inflammation, and apoptosis, especially in brain hippocampus tissue. We also report that Lin may be a therapeutic strategy against Cd-induced neurodegeneration by regulating the 4-HNE/NF-κB signaling pathway.

By altering the tight connections between barrier cells, Cd exposure can harm endothelial, epithelial, and glial cells as well as interfere with the primary structural elements of the blood–brain barrier. As a result, Cd that passes into brain tissue may cause adverse effects [2]. One study showed that Cd exposure caused reactive astrocytosis in hippocampal CA1, CA3, and DG regions [9]. In another study, it was reported that CdCl_2_ administration caused significant damage and loss of hippocampal cells, especially pyramidal cells in the DG region in rats [39]. Similarly, in this study, it was determined that Cd exposure caused neurodegeneration, especially in the CA3 and DG regions of the hippocampus.

ROS-induced enhanced lipid peroxidation is one of the consequences of Cd. Increased MDA level is a significant indication of lipid peroxidation since it is one of the main oxidation products of peroxidised polyunsaturated fatty acids [2]. Another harmful byproduct of lipid peroxidation, 4-HNE, plays a significant role as a mediator in the etiology of illnesses linked to oxidative stress [40]. 4-HNE is involved in a number of cytotoxic processes, including inflammatory damage, apoptosis, and protein dysfunction, and it is triggered by ROS [41]. Nonetheless, 4-HNE functions as a biomarker for oxidative stress and lipid peroxidation [42]. One study reported that Cd exposure decreased hippocampal neurodegeneration and SOD and CAT activity while increasing MDA levels [2]. Consistent with these data, Cd exposure increased oxidative stress parameters (4-HNE and MDA) and decreased antioxidant parameters (CAT, SOD and GSH) in the hippocampus.

These results are in agreement with other studies showing that Cd exposure causes oxidative stress in the hippocampus [8, 39]. Overproduction of ROS, followed by inflammation and death of cholinergic and non-cholinergic neurones in the hippocampus and cerebral cortex, is linked to Cd neurodegeneration [8]. In this regard, Cd ions have the ability to deplete intracellular GSH stores, inhibit cellular antioxidant enzymes, displace redox-active metals (such as Fe^+2^), and interfere with the mitochondria’s oxidative phosphorylation process [43]. However, in an intracerebroventricular-streptozotocin-induced Alzheimer’s model in rats with cognitive impairment, naringenin was found to enhance GSH, glutathione reductase (GR), glutathione peroxidase (GSH-Px), SOD, and CAT to detoxify 4-HNE in the hippocampus [44]. By lowering ROS production and lipid peroxidation in PC-12 cells, trans-resveratrol enhanced the antioxidant defence system and mitigated the cytotoxic reaction of 4-HNE, according to another study [45]. In a different study, it was reported that rosemary extract and peppermint extract (carnosic acid and rosmarinic acid) showed neuroprotective effects by reducing 4-HNE levels in the cerebral cortex and protein carbonyls in the brain hippocampus in an age-related cognitive decline and Alzheimer’s mouse model (SAMP8) [46]. Similarly, in this study, Lin treatment reduced Cd-induced raised hippocampal 4-HNE and MDA levels and increased antioxidant parameters (CAT, SOD and GSH).

CdCl_2_ can stimulate NF-κB activity in the brain of rats [47]. Thus, it is plausible that elevated ROS levels are the primary cause of the NF-κB transactivation seen in the CdCl_2_ hippocampus [21]. In addition, Cd induces NF-κB overactivation in astrocytes for upregulation of markers of inflammation [48]. Astrocyte activation releases proinflammatory cytokines that play an important role in the progression of neuroinflammation in the brain [49]. Consistent with these data, in the present study, an increase in NF-κB level was detected in the hippocampus tissue of the brain due to Cd exposure.

A study reported that DHA supplementation to CdCl_2_-treated rats suppressed oxidative stress and inflammation by regulating NF-κB activation, and as a result, may protect against cognitive function and hippocampal oxidative damage [39]. Likewise, this investigation found that Lin supplementation to Cd-exposed rats decreased hippocampal NF-κB levels. This may be related to the suppression of Cd-induced oxidative damage by Lin supplementation. C-Jun-N-terminal kinase (JNK), which is crucial for oxidative stress-induced cellular death, is one member of the mitogen-activated protein kinase (MAPK) superfamily that is up-regulated by 4-HNE accumulation [50]. Moreover, citri reticulatae viride pericarpium, a traditional medicinal plant in China, has been reported to repair 4-HNE induced inflammatory damage in PC12 cells by inhibiting the phosphorylation of JNK and activation of NF-κB [51]. Similarly, in this study, we suggest that Lin shows a neoprotective effect by decreasing hippocampal 4-HNE and NF-κB levels.

Cd-induced ROS have been shown to activate several different intrinsic and extrinsic apoptotic pathways, including transcription factors and NF-κB, which can trigger neuroinflammation and apoptosis [52]. After neuroinflammation occurs, neurodegeneration advances due to the activation of apoptotic protein markers. In the rat brain, Cd has been demonstrated to continuously activate protein markers linked to neuronal death, including Casp3 [53]. Consistent with these data, in this study, Cd exposure was found to increase Casp3 levels, a proapoptotic marker, in the hippocampus. These results were consistent with previous studies showing an increase in Casp3 immunoreactivity in brain tissue due to Cd exposure [8, 54]. Nevertheless, a study found that trans-resveratrol may lessen apoptotic neurodegeneration by inhibiting oxidative damage and controlling alterations in 4-HNE-induced protein expression and mitochondria-mediated apoptosis indicators (such caspase-3) [45]. Similarly, in this study, Lin showed an anti-apoptotic effect by decreasing the increased hippocampal Casp3 immunoreactivity due to Cd exposure.

Although this current study provides important evidence for the neuroprotective effects of Lin treatment against Cd-induced hippocampal neurodegeneration, the study has some limitations. The first limitation of the study is that it does not include data on hippocampal tissue accumulation and excretion routes (e.g., urine, feces) that could provide information on the pharmacokinetics and potential interactions of Cd and Lin. Another limitation of the study is that the data obtained on Cd-induced neurodegeneration of the hippocampus, which plays a critical role in cognitive processes and memory formation, were not supported by behavioral tests.

In conclusion, Cd exposure induced neurodegeneration, oxidative stress, increased levels of NF-κB and proapoptotic markers that trigger inflammatory reactions in brain hippocampus tissue. However, Cd-induced neurotoxic effects were largely regulated by Lin supplementation. Lin showed neuroprotective effects against Cd-induced hippocampal neurodegeneration by regulating the 4-HNE/NF-κB pathway. The important data obtained in this study reveal the high potential for investigating the cellular pathways or detailed mechanisms affected by Lin in neurotoxic and/or neurodegenerative pathophysiologies.

## Data Availability

The datasets generated and/or analyzed during the current study are available from the corresponding author upon reasonable request.
